# Binarized neural networks converge toward algorithmic simplicity: empirical support for the learning-as-compression hypothesis

**DOI:** 10.3389/fncom.2026.1791546

**Published:** 2026-05-29

**Authors:** Eduardo Y. Sakabe, Felipe S. Abrahão, Alexandre Simões, Esther Colombini, Paula Costa, Ricardo Gudwin, Hector Zenil

**Affiliations:** 1 Faculdade de Engenharia Elétrica e de Computação (FEEC), Universidade Estadual de Campinas (UNICAMP), Campinas, Brazil; 2 Algorithmic Dynamics Lab, King's College London, London, United Kingdom; 3 Centro de Lógica, Epistemologia e História da Ciência (CLE), Universidade Estadual de Campinas (UNICAMP), Campinas, Brazil; 4 Oxford Immune Algorithmics, Oxford University Innovation & London Institute for Healthcare Engineering, London, United Kingdom; 5 Data Extreme Lab (DEXL), National Laboratory for Scientific Computing (LNCC), Petrópolis, Brazil; 6 São Paulo State University (UNESP), Institute of Science and Technology, Sorocaba, Brazil; 7 Instituto de Computação (IC), Universidade Estadual de Campinas (UNICAMP), Campinas, Brazil; 8 Research Department of Biomedical Computing, School of Biomedical Engineering and Imaging Sciences, King's College London, London, United Kingdom; 9 Research Department Digital Twins, School of Biomedical Engineering and Imaging Sciences, King's College London, London, United Kingdom; 10 King's Institute for Artificial Intelligence, King's College London, London, United Kingdom

**Keywords:** algorithmic information theory, binarized neural networks, Block Decomposition Method, learning as compression, Shannon entropy

## Abstract

Understanding and controlling the complexity of neural networks is a central challenge in machine learning, with implications for generalization, optimization, and model capacity. While most approaches rely on entropy-based loss functions and statistical metrics, these measures often fail to capture deeper, causally relevant algorithmic regularities embedded in network structure. We propose a shift toward algorithmic information theory, using binarized neural networks (BNNs) as a first proxy. Grounded in algorithmic probability (AP) and the universal distribution it defines, our approach characterizes learning dynamics through a formal, causally grounded lens. We apply the Block Decomposition Method (BDM), a scalable approximation of algorithmic complexity based on AP, and demonstrate that it more closely tracks structural changes during training than entropy, generally exhibiting stronger correlations with training loss across a wide range of architectures, datasets, and randomized training runs. These results support the view of training in BNNs as a process of algorithmic compression, where learning corresponds to the progressive internalization of structured regularities. In doing so, our work offers a principled estimate of learning progression and suggests a framework for complexity-aware learning and regularization, grounded in first principles from information theory, complexity, and computability.

## 1 Introduction

Understanding the distributional structure of neural network weights from an information-theoretic perspective has driven a range of advances in training efficiency, model compression, and architecture optimization. For example, Susan and Dwivedi (2014) proposed an entropy-based criterion to dynamically adjust the number of hidden neurons, relating increases in weight entropy to growing representational demands. Similarly, Susan et al. (2019) used the stabilization of weight entropy as a stopping criterion during training. Other approaches incorporate entropy directly into the loss function to regularize complexity and encourage more compact representations (Nowlan and Hinton, 1992; Hinton and van Camp, 1993; Molchanov et al., 2017; Wiedemann et al., 2019). Complementarily, post-training compression techniques leverage entropy coding and quantization to reduce model size with minimal impact on accuracy (Han et al., 2016; Choi et al., 2017; Oktay et al., 2020; Wiedemann et al., 2020b,a).

From the universal (algorithmic) coding theorem (see Section 2) within the context of algorithmic information theory (AIT) (Downey and Hirschfeldt, 2010; Chaitin, 2004; Calude, 2002; Li and Vitányi, 2019), these approaches are often grounded in algorithmic probability (Zenil et al., 2019b; Delahaye and Zenil, 2012; Zenil et al., 2018a; Hernández-Espinosa et al., 2025) and universal (Solomonoff) induction (Li and Vitányi, 2019; Kirchherr et al., 1997), such as the Minimum Description Length (MDL) principle (Wallace and Boulton, 1968; Li and Vitányi, 2019; Rissanen, 1986), which posits that the best model is the one that minimizes the total length of two descriptions: the model itself (its parameters or structure) and the data given the model (how well it fits the data). Shannon entropy is widely used in this context because it quantifies the expected bit-length required to encode outcomes from a probabilistic source (Shannon, 1948), directly aligning with MDL. In the case of neural networks, weight entropy serves as a proxy for model complexity, estimating the information needed to represent the network's parameters. Yet, entropy, while widely adopted, captures only statistical variability, overlooking algorithmic and causal structure critical to understanding how neural networks internalize and compress information.

In this work, we adopt a distinction between descriptions that capture underlying generative mechanisms and those that capture only surface-level statistical regularities. We refer to the former as *causally grounded*, as they aim to identify compact generative processes (e.g., programs) that explain how the data is produced, rather than merely describing correlations in observed outcomes. This distinction is reflected in two complementary notions of complexity: *statistics-based complexity*, as captured by entropy, quantifies the distribution of observed patterns, while *algorithmic complexity*, as approximated by methods such as the Block Decomposition Method (BDM) (Zenil et al., 2018a), aims to measure the length of the shortest generative description of those patterns. As a result, algorithmic complexity provides access to underlying structure beyond purely statistical regularities.

AIT offers an encompassing perspective focused on formal-theoretic measures of complexity (particularly, *algorithmic* (*program-size*) *complexity* and *algorithmic probability*) rather than statistical ones, emphasizing the need to capture not just the statistical properties of the data, but also the generative structure (or process) underlying data. Universal induction has been regarded as a theoretical solution to Artificial General Intelligence (AGI) (Hutter et al., 2024), positing that the most intelligent systems are those capable of compressing and generalizing via the shortest explanatory programs (Hernández-Espinosa et al., 2025; Marvin Minsky, 2014). We build on this foundation using the Block Decomposition Method (BDM) (Zenil et al., 2019b, 2018a, 2020, 2019a), a computable and resource effective approximation to the (semicomputable) algorithmic complexity values, to estimate its value in application to the (global) complexity of neural network weights. BDM offers a practical and computable approximation that captures both statistical (at global scales) and algorithmic regularities (at local scales), providing a complexity measure that is more granular and more robust to changes in computation models, programming languages, and feature selection characterization of (irreducible/incompressible) information content than entropy is (Zenil et al., 2018a; Leyva-Acosta et al., 2024; Zenil, 2020). Such an approach aligns more closely with the theoretical underpinnings of intelligence as algorithmic compression and model synthesis (Lavin et al., 2022; Zenil et al., 2019b; Hernández-Espinosa et al., 2025). Because the most powerful implementation of BDM operates on binary representations (even when it can also deal with non-binary objects) and can deal with 2D objects such as weight matrices, we binarize network weights and constrain our experiments to Binarized Neural Networks (BNNs) (Hubara et al., 2016), which enable its direct application.

In our main experiment, we trained binarized Multilayer Perceptrons (MLPs) with varying hidden-layer widths and depths across different datasets, evaluating the correlation between model complexity, measured via BDM and entropy, and training loss across 200 training runs per architecture. Our findings reveal that the Pearson and Spearman correlations between BDM and training loss were generally higher across datasets and architectures than those obtained using entropy, suggesting that BDM may serve as a more effective indicator of model complexity and its relationship with training dynamics (see also further discussion in Section 6). These empirical results support the broader theoretical claim that training in neural networks can be understood as a process of algorithmic compression (Sutskever, 2023), where structure is extracted and encoded in the weights, mirroring the regression/prediction principles proposed e.g., by Solomonoff (Hernández-Espinosa et al., 2025). These insights highlight the limitations of entropy-based approaches and point to the need for complexity-aware learning principles rooted in algorithmic probability (Zenil et al., 2017; Zenil, 2020), such as those introduced in Hernández-Orozco et al. (2021), which applied BDM to guide learning on non-differentiable spaces.

While related work has primarily examined entropy in terms of layer outputs and mutual information, such as those investigating the bottleneck principle (Tishby and Zaslavsky, 2015; Shwartz-Ziv and Tishby, 2017; Butakov et al., 2024), our approach focuses instead on the complexity of the model itself, specifically the distribution and structure of its weights, rather than the dynamics of input-output mappings in activation values across layers. This distinction matters: weight-based complexity reflects the internal representational capacity and structural organization of the model (which is the case we study in this work); whereas layer output-based measures pertain to how data is processed during inference.

Our results suggest, and we argue, that understanding and quantifying such an intrinsic complexity of a model is essential not only for interpretability and regularization, but also for advancing theoretical and practical progress toward general intelligence (Hernández-Espinosa et al., 2025; Marvin Minsky, 2014), where learning must reflect causal inference and universal compression rather than statistical fitting alone.

## 2 Background

### 2.1 Basics concepts and results in algorithmic information theory

The (unconditional prefix) *algorithmic* (Solomonoff-Kolmogorov-Chaitin) *complexity* of a finite string *x*, denoted **K**(*x*), is defined as


$$\text{K}\left(x\right)=\min\left\{|p||\text{U}\left(p\right)=x\right\}=|x^{*}|,$$

where *p* denotes a program running on a universal machine **U** such that **U**(*p*) = *x* is its output, and |*p*| denotes the length of *p*. The program $x^{*}\in {\text{L}}_{U}$ is the shortest such program, satisfying **U**(x^*^) = *x*. The set **L_*U*_** denotes any (prefix-free or self-delimiting) universal programming language running on **U**, and contains all such programs *p*.

Let ${\text{P}}_{\text{U}}\left[x\right]=\underset{\text{U}\left(p\right)=x}{\sum }2^{-|p|}$ denote the *universal a priori probability* of an arbitrary event *x* which can be understood as the probability of randomly generating (by an i.i.d. stochastic process) a prefix-free (or self-delimiting) program *p* that outputs *x*. In other words, the probability that event *x* occurs resulting from the outcome of at least one of all possible computable generative models, formal theories, computer programs, Turing machines, etc.

A computably enumerable (c.e.) semimeasure **m**(·) is said to be *maximal* if, for any other computably enumerable semimeasure μ(·) with domain defined for all possible encoded objects, where $\underset{x\in {\left\{0,1\right\}}^{*}}{\sum }\mu \left(x\right)\leq 1$, there is a constant *C* > 0 (which does not depend on *x*) such that, for every encoded object *x*, **m**(*x*) ≥ *C μ*(*x*).

From the *algorithmic coding theorem* (Chaitin, 2004; Calude, 2002; Li and Vitányi, 2019; Downey and Hirschfeldt, 2010) (or universal coding theorem) we have that


$$\begin{array}{l} \begin{array}{c} \text{K}\left(x\right)=-\log\left({\text{P}}_{\text{U}}\left[x\right]\right)\pm \text{O}\left(1\right)=-\log\left(\text{m}\left(x\right)\right)\pm \text{O}\left(1\right). \end{array} \end{array}$$ (1)

In Equation 1, **O**(1) denotes an additive constant independent of *x*, arising from the choice of the universal machine. We call **m**(*x*) the *algorithmic probability* (AP) of *x*.

### 2.2 The Coding Theorem Method

The Coding Theorem Method (CTM) (Delahaye and Zenil, 2012) is a numerical technique for estimating prefix Kolmogorov complexity by approximating algorithmic probability. The method leverages the relation between **K**(*x*) and **m**(*x*) established by the algorithmic coding theorem, and estimates **m**(*x*) empirically from the output distribution of small programs (here, small deterministic Turing machines).

CTM operates by defining a finite space of deterministic Turing machines with a fixed number of states and a binary alphabet, executing all machines in this space (or a tractable subset of it), and recording the outputs of those that halt. In practice, exhaustive enumeration of *n*-state Turing machines is feasible only for small *n* (typically *n* ≤ 5), since the number of transition tables *N* grows exponentially with the number of states as *N*(*n*) = (4*n* + 2)^2*n*^.

Let *D*_*n*_(*x*) denote the empirical frequency of a binary string *x* among the outputs of all halting *n*-state, two-symbol Turing machines considered. Using the relation between **K**(*x*) and **m**(*x*), CTM substitutes **m**(*x*) with *D*_*n*_(*x*) and approximates


$$CTM\left(x\right)=-\log_{2}D_{n}\left(x\right)\approx \text{K}\left(x\right),$$

up to an additive constant independent of *x*. CTM thus provides an empirical approximation to prefix Kolmogorov complexity for short binary objects. However, due to the combinatorial explosion of the Turing machine space, CTM tables are currently available only for short 1D binary strings (up to 13 bits) and small 2D binary blocks (4 × 4) (Zenil et al., 2015b). For larger objects, the Block Decomposition Method (BDM) extends CTM by decomposing the input into small blocks for which CTM values are available.

### 2.3 The Block Decomposition Method

The Block Decomposition Method (BDM) (Zenil et al., 2019a, 2018a) presents an estimator of algorithmic information redundancies defined by a decomposition of the object into parts for which one already has an algorithmic complexity estimation, obtained by means of, for example, the Coding Theorem Method (CTM) (Delahaye and Zenil, 2012) based on Algorithmic Probability (AP) (see Section 2.1) and the related universal distribution (Kirchherr et al., 1997) which takes into consideration all the statistical and algorithmic regularities and redundancies in data. By finding the smallest generating programs (or models), BDM extends the power of CTM by joining these programs together (in a coarse-graining manner) in order to offer a generative computational model of the object, so that one can always achieve tighter lower bounds on AP (or upper bounds on **K**) by running CTM further. As mentioned in Section 2.1, AP gives an agnostic and invariant probability measure for a randomly generated explanation (e.g., a randomly generated computer program) to explain an object (Kirchherr et al., 1997) or a (-n encodable) set of phenomena so that it is independent (up to a ‘small' constant that has been proven to present a stable rate; Zenil, 2020; Leyva-Acosta et al., 2024) for the chosen computation model, most prominently for low-complexity (or equivalently high algorithmically probable) objects. In addition, it demonstrated to be invariant for an arbitrarily chosen programming language, prior probability distribution, and formal theory in the asymptotic limit as the object size increases.

In general case for any encodable multidimensional object (Ozelim et al., 2025), the BDM of an object *x* is defined by


$$\begin{array}{l} BDM\left(x,i,m\right)=\underset{\left(r_{j},n_{j}\right)\in P_{i}\left(x\right)}{\sum }(\log\left(n_{j}\right)+K_{m}\left(r_{j}\right)), \end{array}$$ (2)

where:

- the partition (to which one assigns the corresponding index *i*) is one of the ways to decompose the object *x*;
- *P*_*i*_(*x*) is the set of pairs (*r*_*j*_, *n*_*j*_) obtained when decomposing the object *x* according to a partition *i* of contiguous parts *r*_*j*_, each of which appears *n*_*j*_ times in such a partition (i.e., *n*_*j*_ is the multiplicity of *r*_*j*_), that is, the number of exact repetitions;
- *K*_*m*_(*r*_*j*_) is an approximation to **K**(*r*_*j*_) and *m* is the index of the approximation method employed to calculate *K*_*m*_(*r*_*j*_).

Equation 2 can be expressed for unidimensional objects but also to encodable multidimensional objects in general, such as non-binary strings and *n*-dimensional objects such as graphs, matrices, images, vectors and tensors (Zenil et al., 2018a, 2019a; Zenil, 2020; Zenil et al., 2018b). For example, for a bit string *x*, Equation 2 holds for a partition defined by the sequence of contiguous linear blocks (of length ≥1) whose concatenation reconstructs *x*.

From classical information theory, we have that


$$\begin{array}{l} {\text{H}}_{i}\left(X^{\left(i\right)}\right)=-\underset{\left(r_{j},n_{j}\right)\in P_{i}\left(x\right)}{\sum }p\left(r_{j}\right)\log\left(p\left(r_{j}\right)\right) \end{array}$$ (3)

is the *block* (Shannon) entropy **H**_*i*_ of an i.i.d. source *X*^(*i*)^ such that $p\left(r_{j}\right)\to \frac{n_{j}}{N_{i}}$ as |*x*| → ∞, the random variable *X*^(*i*)^ can assume values in the set {*r*_1_, …, *r*_*j*_, …, *r*_|_*P*__*i*_(*x*)|_}, and $N_{i}=\left(\underset{\left(r_{j},n_{j}\right)\in P_{i}\left(x\right)}{\sum }n_{j}\right)$.

Thus, **H** is a basis for (statistical) compression methods that are subsumed into BDM while for sufficiently large objects both converge to each other. This is because BDM characterizes the information content of the entire object by adding the estimated (local) complexity given by **K** and the (global) Entropy (**H**) values as described in Equation 2 (Ozelim et al., 2025; Zenil et al., 2018a).

Our results in this paper corroborate these mathematical properties of BDM and entropy, the former expected to perform better for smaller objects while being more sensitive to patterns other than statistical ones. In Section 5.3, our control experiments evince the case in which both are indeed expected to converge.

## 3 Measuring the complexity of binarized neural networks

Our primary objective is to investigate whether algorithmic complexity, estimated via the Block Decomposition Method (BDM), serves as a more informative indicator of neural network learning dynamics than entropy. We hypothesize that training a neural network reduces the algorithmic complexity of its weights by aligning them with the structural regularities of the data. In this framing, learning is understood as a form of algorithmic compression: transforming initially random, high-complexity parameters into structured configurations that encode the input-output mappings required by the task.

Accordingly, we expect BDM, which captures local algorithmic regularities beyond statistical variability, to correlate more strongly with training loss than entropy does. While entropy quantifies the expected bit-length under a probabilistic model, it does not account for causal or generative structure within the weight matrix. In contrast, BDM, grounded in algorithmic information theory, approximates algorithmic complexity by detecting repeatable, low-complexity patterns, even in systems that may appear statistically random.

Furthermore, alternative complexity measures based on compression, such as the widely used Lempel-Ziv-Welch (LZW) (Ziv and Lempel, 1977; Welch, 1984), primarily capture statistical regularities through pattern frequencies and therefore exhibit behavior closely aligned with entropy (Zenil, 2020). By contrast, BDM leverages the Coding Theorem Method (CTM), providing a principled approximation of algorithmic complexity that is sensitive to underlying generative structure beyond statistical distributions.

This distinction also informs our experimental design. As discussed in Section 2.3, BDM converges toward entropy for large objects; accordingly, we focus on small binarized MLPs where it remains sensitive to algorithmic structure. Within this regime, we can meaningfully evaluate whether learning corresponds to a process of algorithmic compression, rather than purely statistical compression.

This hypothesis builds on the assumption that effective learning involves the internalization of data structure into the model's parameters in a compact, structured form. We take the training loss as a proxy for this process, assuming that as the loss decreases, the network is increasingly aligned with the regularities in the training data. However, because low loss can also result from memorization rather than generalization, we constrain our analysis to the training regime before the onset of overfitting, as indicated by a plateau in validation loss–where the model is likely compressing the data's functional structure rather than encoding idiosyncratic details of the training data.

Our approach is consistent with the Minimum Description Length (MDL) principle (Wallace and Boulton, 1968; Rissanen, 1986), and related perspectives in deep learning that frame training as a compression process (Tishby and Zaslavsky, 2015; Schmidhuber, 1997). In particular, we focus on the complexity of the model parameters as a proxy for the model description component of MDL, rather than the data-given-the-model (functional) description. We then compare statistical and algorithmic measures of this quantity. By directly comparing BDM and entropy under identical training conditions, we aim to evaluate whether BDM better captures meaningful structural transformations in the model's parameters during learning.

This view of training as a form of algorithmic compression is supported by recent commentary by Sutskever (2023), who suggests that gradient-based optimization, particularly Stochastic Gradient Descent (SGD), can be seen as an implicit algorithmic search, uncovering compressed programs within the neural network's weights.

### 3.1 Computing BDM and entropy in binarized neural networks

To assess the complexity of a fully connected binarized neural network during training, we compute two measures over its binarized weight matrices: algorithmic complexity using the Block Decomposition Method (BDM), and statistics-based complexity using entropy. Both measures are derived from a common decomposition of the weights into fixed-size binary submatrices.

#### 3.1.1 Weight extraction and binarization

We extract all weight matrices from the model, excluding batch normalization layers. Each matrix is binarized by applying the sign function, mapping values >0 to 1 and ≤ 0 to 0. This produces a set of 2D binary matrices, suitable for pattern-based analysis.

#### 3.1.2 Shared block decomposition

Each binarized matrix is partitioned into non-overlapping 4 × 4 blocks. This yields a multiset of binary patterns used as atomic units for both BDM and entropy computation. For each matrix, we count the occurrences of each unique 4 × 4 block. These counts define an empirical distribution over the observed patterns.

#### 3.1.3 Shannon entropy

The entropy of a matrix is computed using the empirical distribution of 4 × 4 patterns as defined in Equation 3. Here, *p*(*r*_*j*_) corresponds to the relative frequency of pattern *r*_*j*_ across all blocks. This entropy captures the statistical variability of local structures in the network's weights.

#### 3.1.4 Block Decomposition Method (BDM)

To estimate algorithmic complexity, we apply BDM as defined in Equation 3. Each unique 4 × 4 block *r*_*j*_ is assigned a complexity value based on the Coding Theorem Method (CTM), and repeated occurrences are penalized logarithmically. This yields a composite complexity score reflecting both diversity and compressibility of local patterns.

All computations, including block partitioning, empirical distribution estimation, entropy, and BDM values, were implemented using the pybdm library (Talaga and Tsampourakis, 2024).

## 4 Experiments

We evaluated the relationship between model complexity and learning dynamics in binarized neural networks across a range of datasets. To assess the robustness of our findings, we also included two control settings: one in which MNIST labels were randomly permuted, and another in which both inputs and labels were random noise. These controls allow us to disentangle effects driven by genuine data structure from those arising in the absence of a learnable signal.

This experimental setup is guided by the constraints of the complexity measure and model class: because BDM remains informative primarily in small-scale settings, we employ low-capacity binarized neural networks, which in turn necessitates the use of datasets whose underlying structure can be effectively captured within these models.

### 4.1 Datasets

We evaluated our approach on a diverse set of relatively simple datasets, compatible with our low-capacity models, spanning image classification, human activity recognition, and controlled synthetic settings. This selection allows us to study learning dynamics across varying degrees of statistical and structural regularity within the representational constraints of the models.

**Figure 1:**
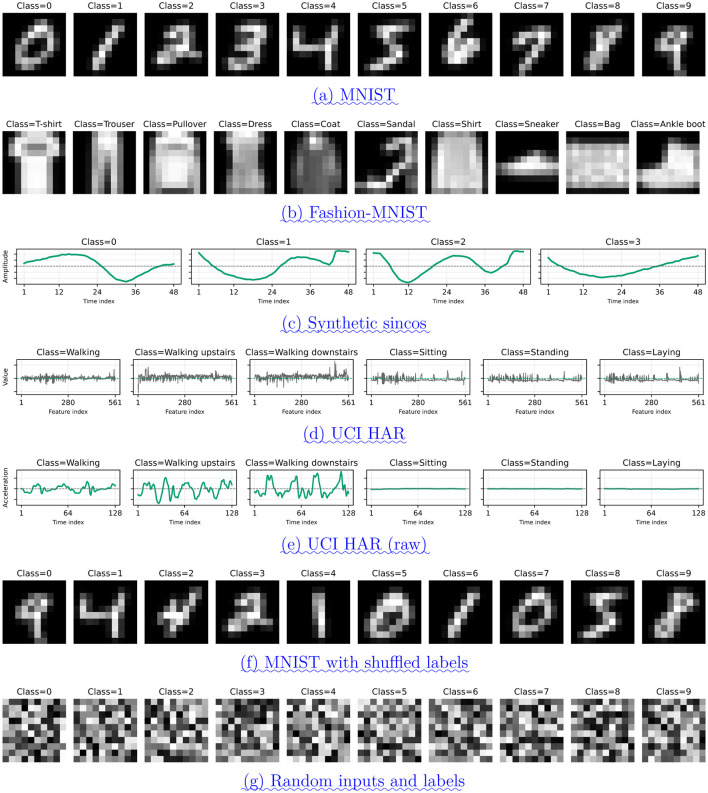
Representative input–output examples for the datasets used in the experiments. Panels **(a)** and **(b)** show resized 10 × 10 grayscale image examples from MNIST and Fashion-MNIST, respectively. Panel **(c)** shows one-dimensional synthetic sincos signals generated from class-dependent sine–cosine mixtures. Panel **(d)** shows standardized 561-dimensional engineered feature vectors from UCI HAR. Panel **(e)** shows a representative standardized inertial time-series signal from UCI HAR (raw), specifically the body acceleration along the *x*-axis (body_acc_x) over 128 time steps. Panels **(f, g)** show the two control conditions: MNIST inputs paired with shuffled labels and random 10 × 10 inputs paired with random labels.

Figure 1 shows representative input–output examples for each dataset included in our experiments.

#### 4.1.1 MNIST (baseline)

We used the MNIST dataset of handwritten digits (Lecun et al., 1998) as the baseline benchmark in our experiments. MNIST consists of 28 × 28 grayscale images of digits from 0 to 9. Images were resized to 10 × 10 pixels to reduce input dimensionality and to avoid regimes in which BDM approximations collapse to entropy-like behavior. Pixel values were normalized using the dataset mean and standard deviation.

#### 4.1.2 Fashion-MNIST

This dataset is a drop-in replacement for MNIST (Xiao et al., 2017), consisting of 28 × 28 grayscale images of clothing items across 10 categories. As with MNIST, images were resized to 10 × 10 pixels and normalized using dataset statistics. This dataset provides a more visually complex alternative while retaining comparable dimensionality and class structure.

#### 4.1.3 Synthetic sincos dataset

We additionally devised a synthetic classification dataset, referred to as *sincos*, constructed from mixtures of sinusoidal and cosinusoidal signals with class-dependent parameters. Each example consists of a one-dimensional signal of length 48, generated as a sum of a sine and a cosine component with class-specific amplitudes, frequencies, and phase offsets. The dataset comprises four classes, each corresponding to a distinct parameter configuration. To introduce controlled variability while preserving the underlying generative structure, signals were subject to small random perturbations, including phase and amplitude jitter, minor frequency variations, circular shifts, smooth time warping, and additive Gaussian noise. Despite the shared “sincos” terminology and use of sinusoidal components, this dataset differs from prior sincos benchmarks (Yadav et al., 2025; Yadav and Stender, 2025) in that it defines a classification problem over stochastic signal realizations rather than a deterministic signal-to-signal mapping.

#### 4.1.4 UCI HAR

The UCI Human Activity Recognition (HAR) dataset (Anguita et al., 2013) consists of multivariate time-series sensor data collected from a smartphone accelerometer and gyroscope, labeled with six activities: walking, walking upstairs, walking downstairs, sitting, standing, and lying. We used the preprocessed version provided by the dataset authors, in which fixed-length windows of raw tri-axial accelerometer and gyroscope signals are transformed into engineered feature vectors.

The feature representation includes time- and frequency-domain descriptors computed from body and gravity acceleration signals, angular velocity signals, and their first temporal derivatives. Examples include statistical measures such as mean, standard deviation, signal magnitude area, energy, entropy, and correlation, as well as frequency-domain attributes derived from the Fast Fourier Transform. Each input example is represented by a fixed 561-dimensional feature vector summarizing sensor statistics over a temporal window.

#### 4.1.5 UCI HAR (raw)

To contrast with the engineered-feature setting, we also used the raw version of the UCI HAR dataset, retaining the original tri-axial accelerometer and gyroscope signals prior to feature extraction. This variant preserves the temporal structure of the sensor measurements rather than aggregated statistics. Each input example is represented as a 1,152-dimensional vector corresponding to a fixed-length multivariate time series. This comparison allows us to assess whether feature engineering affects the relationship between learning dynamics and complexity measures.

#### 4.1.6 Control datasets

To assess whether observed correlations between model complexity and training loss depend on meaningful input–output structure, we included two control settings. In the first, MNIST labels were randomly permuted while inputs were kept unchanged, preserving the input distribution but destroying semantic correspondence. In the second, we used a fully synthetic dataset consisting of 10 × 10 inputs with entries sampled independently and uniformly from [0, 1), paired with randomly assigned class labels. In both cases, learnable structure is absent by construction, such that any apparent learning reflects memorization rather than compression of meaningful patterns. These controls serve as baselines for interpreting complexity dynamics in the absence of genuine structure.

To evaluate generalization and monitor overfitting, we applied a consistent data-splitting protocol across all datasets. For each dataset, we constructed a stratified validation set from the training data and used the remaining examples as a training pool. We then halved this pool via stratified sampling to generate 200 independent training subsets, preserving class proportions and ensuring disjointness from the validation set. Each subset trained a separate model instance.

We illustrate the protocol using MNIST as a representative example. From the original 60,000-example training set, we constructed a stratified validation set of 10,000 examples. The remaining 50,000 examples formed a training pool, which we further halved to produce 200 stratified subsets of 25,000 examples each. We held out the standard MNIST test set (10,000 examples) and the corresponding test sets for other datasets exclusively for reporting final test accuracy.

### 4.2 Model architecture

In all experiments, we used binarized Multilayer Perceptrons (MLPs) (Hubara et al., 2016), where we binarized both weights and activations and trained them using the Straight-Through Estimator (STE) (Bengio, 2013) to enable backpropagation through discrete operations. We applied batch normalization after each hidden layer to stabilize training.

We varied MLP architectures across datasets. We tested models with one to four hidden layers, choosing layer widths to enable comparisons at comparable total parameter counts while accounting for differences in input and output dimensionality across datasets.

### 4.3 Training procedure

For each dataset and model architecture, we trained 200 independent model instances on different stratified subsets of the training pool (Section 4.1). We employed early stopping based on validation loss, selecting the checkpoint with the lowest validation loss and stopping training when it failed to improve for a configuration-specific patience.

We optimized the cross-entropy objective using the Adam optimizer. Learning rates, mini-batch sizes, and early-stopping patience varied across configurations; we report all hyperparameter values in the Supplementary Table S1.

During training, we evaluated BDM, Shannon entropy, and training loss at every optimization step, and evaluated the validation loss every 20 steps. We then averaged all quantities per epoch to enable direct comparison between complexity and loss trajectories.

All experiments were run in PyTorch with Metal acceleration on a MacBook Pro (M4 Max). For the largest architectures, training and complexity analysis across 200 runs required approximately 15 hours.

### 4.4 Evaluation

We assessed the relationship between model complexity and learning dynamics by analyzing the correlations between mean training loss and mean complexity metrics (BDM and entropy) computed per epoch throughout training. Our evaluation followed three main steps.

#### 4.4.1 Metric normalization

To ensure comparability across metrics and reduce noise, we applied a normalization pipeline to the per-epoch series of training loss, BDM, and entropy values. We excluded the final *patience* epochs prior to early stopping to avoid the overfitting regime. Each metric was normalized independently for each run using: (i) a log transformation (np.log1p) to reduce skewness, (ii) Gaussian smoothing with σ = 1 to attenuate high-frequency noise, and (iii) Min–Max scaling to the [0, 1] range to enable consistent comparisons across metrics and runs.

#### 4.4.2 Correlation analysis

Using the normalized values, we computed Pearson and Spearman correlation coefficients between training loss and each complexity metric. These correlations quantified both linear (Pearson) and monotonic (Spearman) relationships, providing insight into how closely each metric tracked the progression of learning.

#### 4.4.3 Bootstrap confidence intervals

To assess statistical reliability, we estimated 95% confidence intervals via bootstrap resampling over the 200 independently trained models for each architecture. This provided robust estimates of variability for all reported correlations.

## 5 Results

We present the experimental results in this section. The majority of configurations retain the full set of 200 independent runs. In a small number of cases, primarily among smaller models, early stopping produces runs shorter than five training epochs. Such runs are excluded from the correlation analysis, as they do not provide a sufficient number of training epochs for reliable correlation estimation, resulting in reduced sample sizes for these configurations. Test accuracies are also reported to verify that all non-control models achieve performance above random chance. Representative model configurations are shown in the main text, while the Supplementary Table S2 provides the complete set of results across all evaluated architectures (50 in total), spanning MNIST (14), Fashion-MNIST (5), *sincos* (14), UCI HAR (5), UCI HAR (raw) (2), and control conditions comprising MNIST with shuffled labels (5) and fully random inputs with random labels (5). The table additionally reports the effective sample sizes used in the bootstrap analysis for each configuration.

### 5.1 MNIST results (baseline)

Table 1 reports the results for MNIST using standard two-hidden-layer MLPs of increasing capacity. The table shows 95% confidence intervals for Pearson (*r*) and Spearman (ρ) correlations between model complexity (measured using BDM and Shannon entropy) and the mean training loss. For each configuration, we also report the total number of trainable parameters and the final test accuracy of the model selected according to the lowest validation loss.

**Table 1:** Correlation [95% CI] between training loss and complexity metrics on MNIST for two-hidden-layer MLP architectures of increasing capacity.

Hidden sizes	Params	Entropy *r*	BDM *r*	*Δr*	Entropy ρ	BDM ρ	*Δρ*	Acc. (%)
8, 4	872	[0.47, 0.60]	**[0.77, 0.86]**	0.28	[0.52, 0.68]	**[0.74, 0.85]**	0.19	52.1 ± 5.3
16, 8	1808	[0.55, 0.65]	**[0.90, 0.93]**	0.31	[0.58, 0.70]	**[0.85, 0.91]**	0.24	67.9 ± 2.4
32, 16	3872	[0.74, 0.79]	**[0.92, 0.94]**	0.16	[0.73, 0.81]	**[0.84, 0.90]**	0.10	79.5 ± 1.0
64, 32	8768	[0.84, 0.86]	**[0.93, 0.94]**	0.09	[0.85, 0.89]	[0.89, 0.92]	0.04	85.9 ± 0.5
128, 64	21632	[0.86, 0.87]	**[0.92, 0.93]**	0.06	[0.92, 0.94]	[0.93, 0.95]	0.01	89.0 ± 0.4

*r* and ρ denote Pearson and Spearman correlations, respectively. Δ*r* and Δρ report the difference between midpoint estimates of BDM and entropy correlations [midpoint (BDM) minus midpoint (entropy)]. Bold values indicate cases of non-overlapping confidence intervals, with the higher correlation highlighted. Accuracy is reported as mean ± standard deviation.

The complete set of configurations, including additional architectures, is reported in Supplementary Table S2.

Across all two-layer configurations, correlations between training loss and BDM are consistently higher than those measured using entropy for both Pearson and Spearman statistics. The magnitude of this difference decreases monotonically as model capacity increases, as reflected by the midpoint differences Δ*r* and Δρ. As model size grows, entropy-based correlations increasingly approach those obtained with BDM.

**Figure 2:**
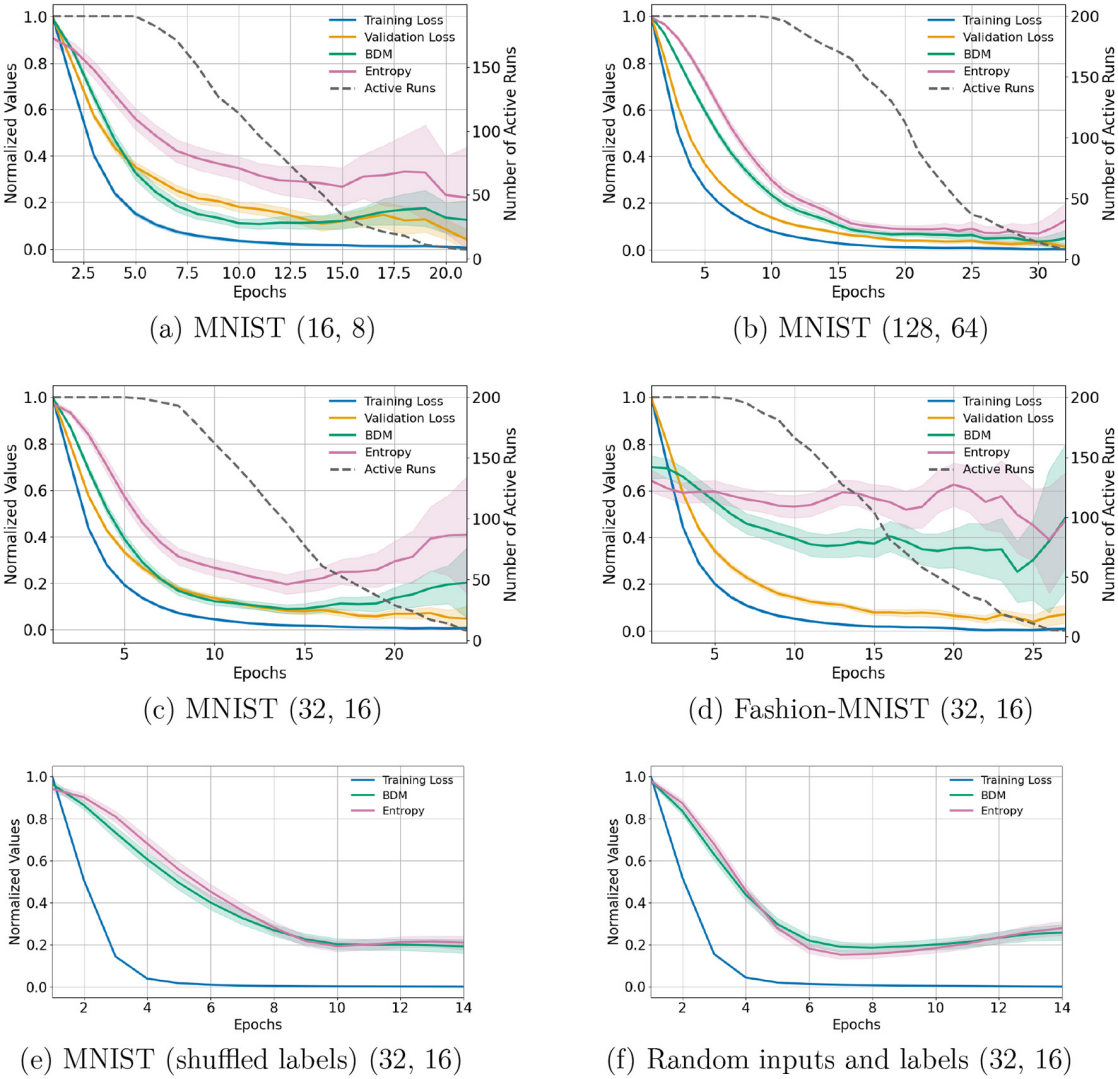
Evolution of training loss, validation loss, BDM, and Shannon entropy across training epochs for different model architectures and datasets. Panels **(a–c)** show MNIST models with increasing hidden-layer sizes, panel **(d)** shows Fashion-MNIST, and panels **(e, f)** show control conditions with shuffled labels and fully random inputs, respectively, all using the (32, 16) architecture. All quantities are normalized to facilitate comparison across runs and metrics. Results are based on 200 independent training runs per configuration. Shaded regions denote 95% confidence intervals, computed only for epochs with at least five active runs. The dashed gray curve indicates the number of active runs per epoch, reflecting the effect of early stopping. For MNIST, BDM more closely tracks the training loss than entropy across architectures, with the difference most pronounced for smaller models. For Fashion-MNIST, the alignment between complexity measures and training loss is weaker overall, though BDM remains more closely aligned than entropy. In the random-data control conditions, the difference between BDM and entropy largely vanishes.

Figures 2a–c shows the corresponding MNIST training dynamics. Across configurations, BDM more closely tracks the training loss than entropy, while entropy exhibits weaker alignment and greater variability across runs.

To assess whether these trends depend on network depth rather than model capacity, Table 2 reports results for MNIST architectures with varying numbers of hidden layers but comparable total parameter counts. Across these configurations, the comparative relationship between BDM and entropy is consistent with that observed for two-layer networks.

**Table 2:** Correlation [95% CI] between training loss and complexity metrics on MNIST for MLP architectures with varying depth and comparable total parameter counts.

Depth	Hidden sizes	Params	Entropy *r*	BDM *r*	Entropy ρ	BDM ρ	Acc. (%)
1	80	8800	[0.84, 0.86]	**[0.92, 0.93]**	[0.83, 0.88]	**[0.89, 0.93]**	88.5 ± 0.4
2	64, 32	8768	[0.84, 0.86]	**[0.93, 0.94]**	[0.85, 0.89]	[0.89, 0.92]	85.9 ± 0.5
3	40, 64, 32	8928	[0.77, 0.80]	**[0.91, 0.93]**	[0.71, 0.79]	**[0.80, 0.86]**	83.7 ± 0.7
4	32, 64, 44, 16	8928	[0.66, 0.73]	**[0.87, 0.90]**	[0.67, 0.76]	**[0.80, 0.86]**	79.4 ± 1.0

Metrics and notation are as in Table 1. Bold values indicate cases of non-overlapping confidence intervals, with the higher correlation highlighted.

### 5.2 Results across datasets

Table 3 reports results across datasets using two-hidden-layer MLPs with comparable parameter counts. For each dataset, we report correlations between training loss and model complexity measured using BDM and Shannon entropy, together with final test accuracy.

**Table 3:** Correlation [95% CI] between training loss and complexity metrics across datasets using two-hidden-layer MLPs with comparable parameter counts.

Dataset	Hidden sizes	Params	Entropy *r*	BDM *r*	Entropy ρ	BDM ρ	Acc. (%)
mnist	32, 16	3872	[0.74, 0.79]	**[0.92, 0.94]**	[0.73, 0.81]	**[0.84, 0.90]**	79.5 ± 1.0
fashion mnist	32, 16	3872	[0.03, 0.17]	**[0.30, 0.46]**	[0.04, 0.18]	**[0.32, 0.48]**	73.1 ± 0.9
sincos	64, 32	5440	[0.36, 0.45]	**[0.71, 0.77]**	[0.45, 0.55]	**[0.77, 0.83]**	96.5 ± 0.7
uci har	8, 4	4544	[0.68, 0.77]	[0.63, 0.74]	[0.57, 0.68]	[0.51, 0.63]	73.7 ± 6.5
uci har (raw)	4, 16	4768	[0.57, 0.70]	[0.49, 0.64]	[0.54, 0.66]	[0.46, 0.60]	50.9 ± 2.6

Metrics and notation follow Table 1. Bold values indicate cases of non-overlapping confidence intervals, with the higher correlation highlighted.

Compared to the MNIST baseline, Fashion-MNIST exhibits substantially lower correlations for both complexity measures at comparable model capacity, a trend also visible in Figure 2d. Despite this reduction, correlations based on BDM remain higher than those obtained using entropy for both Pearson and Spearman statistics.

The synthetic *sincos* dataset displays patterns similar to MNIST, with strong correlations between training loss and both complexity measures and consistently higher correlations for BDM than for entropy.

In contrast, for the UCI HAR dataset, correlations obtained using entropy are similar to or slightly higher than those obtained using BDM. The same pattern is observed for the raw-signal variant of UCI HAR, where no consistent advantage of BDM over entropy is apparent.

Complete results for a broader range of network widths and depths across all datasets are reported in the Supplementary Table S2.

### 5.3 Control experiments

For the control experiments, correlations were computed over a fixed window of 14 training epochs, corresponding to the mean epoch of best validation performance (early stopping) observed across the 200 MNIST (32, 16) runs. In contrast to the main experiments, control models were trained for a fixed duration of 14 epochs without early stopping, since validation loss is uninformative in the absence of shared structure between training and validation sets.

Table 4 summarizes results for baseline and control datasets using a fixed two-hidden-layer architecture. While MNIST exhibits a consistent advantage of BDM over entropy in terms of correlation with training loss, this effect does not extend to the control settings. For shuffled-label MNIST and the dataset with fully random inputs and labels, correlations obtained using BDM and entropy are comparable. This absence of separation is also reflected in the training dynamics shown in Figures 2e, f. The same qualitative outcome is observed across different architectural configurations, as confirmed by additional experiments reported in the Supplementary Table S2.

**Table 4:** Correlation [95% CI] between training loss and complexity metrics for baseline and control datasets using a fixed two-hidden-layer MLP architecture (32, 16).

Dataset	Entropy *r*	BDM *r*	*Δr*	Entropy ρ	BDM ρ	*Δρ*
mnist	[0.74, 0.79]	**[0.92, 0.94]**	0.16	[0.73, 0.81]	**[0.84, 0.90]**	0.10
mnist_shuffled	[0.60, 0.66]	[0.65, 0.71]	0.05	[0.67, 0.75]	[0.67, 0.75]	0.00
random	[0.75, 0.80]	[0.74, 0.79]	–0.01	[0.48, 0.57]	[0.50, 0.61]	0.03

Metrics and notation follow Table 1. Bold values indicate cases of non-overlapping confidence intervals, with the higher correlation highlighted.

## 6 Discussion

We first verified that all models performed significantly above chance across all datasets. On MNIST, where a random classifier achieves roughly 10% accuracy, even the smallest architecture exceeded 50%, indicating that the networks learned meaningful input–output mappings from the data. Similar results were observed for the remaining datasets, with all architectures, except for the random control models, achieving accuracy above 50%.

The analysis below is based on the representative configurations presented in the previous section and is further corroborated by the complete set of results reported in the Supplementary Table S2, which exhibit trends consistent with our findings across datasets and architectural variations.

### 6.1 Interpretation of MNIST loss–complexity correlations (baseline)

The correlation results on MNIST reveal strong positive correlations between model complexity and training loss for both BDM and entropy. These findings indicate that as models reduce error over time, their structural and statistical complexity also decrease. Across all MNIST configurations, BDM exhibits higher Pearson and Spearman correlations than entropy, consistent with greater sensitivity to training-driven changes in model structure, particularly in smaller architectures where algorithmic regularities are more pronounced. This correlation advantage of BDM over entropy remains consistent across architectures with different network depths but comparable parameter counts.

The higher Pearson correlations imply that BDM tracks the magnitude of changes in training loss more closely. Concurrently, the stronger Spearman correlations indicate that BDM better preserves the relative ordering of complexity over training epochs. Together, these results suggest that BDM provides a richer signal of learning progression than entropy, likely due to its foundation in algorithmic information theory, which captures more than just statistical regularities (see Section 2).

However, this advantage diminishes as model size increases, since BDM relies on evaluating fixed-size 4 × 4 binary blocks via the CTM. As the size of the network grows, the decomposition process leads to increasing redundancy and a heavier influence of the multiplicity term log_2_*n*_*i*_ in Equation 2. This results in a loss of granularity and a convergence of BDM toward entropy-like behavior, reducing its ability to discriminate structural complexity. Consequently, in larger models, BDM transitions from an algorithmic to a more statistical measure (see Section 2 and a discussion on limitations in Section 6.4).

The training dynamics shown in Figures 2a–c further support these findings. Across all architectures, BDM exhibits a trajectory that more closely follows the evolution of training loss compared to entropy. This temporal alignment reinforces the view that BDM is more responsive to the structural transformations that occur as the model learns. Moreover, although confidence intervals naturally widen toward later epochs due to early stopping and reduced sample sizes, entropy displays greater variability across runs at each epoch. This difference in variance suggests that BDM not only correlates more strongly with training loss but also produces more stable complexity estimates during training.

### 6.2 Interpretation of loss–complexity correlations across datasets

The results on the *sincos* and Fashion-MNIST datasets largely reinforce the trend observed on MNIST. In both cases, correlations between training loss and model complexity remain higher when measured using BDM than when measured using entropy, indicating a consistent relative advantage of BDM across datasets.

At the same time, Fashion-MNIST exhibits substantially lower absolute correlations for both complexity measures compared to MNIST, with correlations that are no longer strongly positive. While the precise cause of this reduction is not fully clear, one plausible explanation is that Fashion-MNIST constitutes a more challenging learning task, limiting the extent to which the model can progressively compress task-relevant structure during training. Under such conditions, both algorithmic and statistical complexity measures may provide weaker alignment with training loss.

In contrast to MNIST, Fashion-MNIST, and *sincos*, the UCI HAR dataset does not exhibit a clear advantage of BDM over entropy, despite achieving a non-trivial classification accuracy. This suggests that successful task performance alone is not sufficient to induce stronger algorithmic complexity–loss correlations. One initial hypothesis was that the engineered statistical features used in UCI HAR may suppress regularities that could otherwise be captured and compressed by the network. To test this possibility, we repeated the analysis using the raw-signal variant of UCI HAR under comparable model capacity. However, no consistent advantage of BDM over entropy was observed in this setting either.

At present, we do not have a definitive explanation for this behavior. One plausible interpretation is that, for UCI HAR, the predictive input–output relationship is dominated by statistical regularities that are not well reflected as compressible algorithmic structure at the level captured by our weight-based BDM estimates. In such cases, entropy-based measures may be sufficient to capture relevant complexity changes during training. Additional experiments with alternative network widths and depths, reported in the Supplementary material, yielded qualitatively similar results. It is also possible that the relatively high dimensionality of the raw UCI HAR inputs limits the ability of the current models to effectively compress structural information. Increasing model capacity, using the regime observed in the MNIST case as a reference, would likely place BDM in a setting where its estimates increasingly resemble entropy, thereby reducing its discriminative advantage.

These observations are consistent with the full set of results reported in Supplementary Table S2.

### 6.3 Validation of loss–complexity correlations through control experiments

In both control experiments, the models successfully minimized training loss over the fixed window of 14 epochs, demonstrating their capacity to memorize data even in the absence of learnable structure. This holds both when the input–output mapping is destroyed, as in shuffled-label MNIST, and when both input structure and labels are fully randomized. Under these conditions, correlations between training loss and model complexity are similar for BDM and entropy across both Pearson and Spearman statistics, with BDM correlations notably reduced.

This contrast with the MNIST baseline reinforces the interpretation that BDM is specifically sensitive to compressible structure in the learned mapping, whereas entropy primarily reflects general memorization dynamics. In the absence of algorithmic regularities, BDM converges toward entropy-like behavior and loses its relative advantage. As shown in Figures 2e, f, the trajectories of BDM and entropy closely align in both control settings, reflecting the lack of structural learning signals and supporting the view that BDM provides additional information only when learning involves meaningful algorithmic structure. Taken together, to our knowledge, this is the first explicit empirical framing of the distinction between learning and memorization in terms of structural (algorithmic) vs. statistical compression.

### 6.4 Regime-dependent behavior and methodological limitations

#### 6.4.1 Regime-dependent behavior

An important outcome of our analysis is that the relationship between BDM, entropy, and training dynamics is not uniform across datasets and model configurations. Rather than constituting a limitation, this variability reflects theoretically grounded boundary conditions under which algorithmic complexity measures are informative.

Across MNIST, Fashion-MNIST, and the synthetic *sincos* dataset (Sections 6.1, 6.2), BDM provides a stronger signal of training dynamics in regimes where models effectively internalize compressible structure. In these settings (Supplementary Table S2), BDM exhibits consistently higher midpoint correlations with training loss than entropy in a large majority of cases, with non-overlapping bootstrap confidence intervals.

This advantage diminishes in three identifiable regimes:

1. *Limited model capacity relative to task complexity* (Fashion-MNIST, UCI HAR raw): When model capacity is insufficient, neither BDM nor entropy exhibits strong correlation with training loss, as the network cannot effectively compress the underlying structure. This is reflected in both reduced accuracy and weaker correlations. In our experiments, this regime is most evident in smaller architectures for Fashion-MNIST and across architectures in UCI HAR raw (see Supplementary Table S2), where the high input dimensionality constrains the use of larger hidden layers and prevents entry into the regime in which BDM approaches entropy.
2. *Large model regimes*: Consistent with theoretical and empirical results on algorithmic complexity (Zenil, 2020), BDM converges toward entropy as model size increases. This behavior arises from the growing dominance of the multiplicity term in the BDM formulation (see Section 6.1), which reduces sensitivity to larger-scale structures. As a result, BDM becomes less responsive to structural regularities at scale, diminishing its discriminative advantage. This trend is reflected in our empirical results, where the correlation advantage of BDM over entropy decreases with model size across MNIST (Table 1), Fashion-MNIST, and *sincos*, as further detailed in Supplementary Table S2.
3. *Statistically dominated datasets* (UCI HAR): In this setting, no consistent advantage of BDM over entropy is observed (Section 6.2). A theory-guided hypothesis is that the predictive structure is primarily statistical rather than algorithmically compressible at the level captured by current BDM estimates. For UCI HAR, this is consistent with the dataset's construction from engineered statistical features derived from raw signals, which may remove algorithmic regularities that could otherwise be captured by the model.

Taken together, these regimes indicate that the behavior of BDM is not task-specific, but instead systematically governed by the interaction between model capacity, data structure, and the limits of current algorithmic complexity approximations.

Importantly, even in settings where BDM does not outperform entropy (UCI HAR and UCI HAR raw), statistically significant differences are rare, with only a single configuration exhibiting non-overlapping confidence intervals (see Supplementary Table S2). Moreover, in both datasets, results closely align with those of the randomized control groups (Section 6.3), suggesting that BDM either captures algorithmic structure when present or converges toward entropy-like behavior without degrading below the baseline established by random controls.

#### 6.4.2 Methodological limitations and future work

The present study is subject to methodological constraints that delimit the scope and applicability of our analysis:

1. *Restriction to binarized representations*. The current implementation of BDM operates on binary two-dimensional structures, restricting our analysis to Binarized Neural Networks (BNNs). While real-valued parameters can, in principle, be represented via binary expansions without altering their Kolmogorov complexity up to additive constants (Staiger, 2002), such representations substantially increase object size, which directly affects BDM, as discussed in the second limitation below.
2. *Scalability of BDM approximations*. As previously stated, BDM relies on precomputed CTM values over 4 × 4 binary blocks, which limits its applicability to larger objects by restricting the capture of algorithmic structure to local patterns. As a result, in larger systems, global structure is increasingly approximated through the multiplicity term. Extending BDM to larger-scale systems (e.g., larger, practical neural networks) would require substantially larger CTM blocks. However, the size of the underlying Turing machine space grows combinatorially with the number of states and symbols, making such extensions computationally demanding in practice (Zenil et al., 2015b).
3. *Non-differentiability of algorithmic complexity*. Algorithmic complexity is inherently non-continuous and non-differentiable, preventing its direct integration into gradient-based optimization methods such as backpropagation. Consequently, BDM cannot be directly employed as a training-time regularizer.

Together, these constraints limit the direct applicability of our approach to large-scale, non-binarized neural networks. Developing scalable and differentiable approximations of algorithmic complexity, or extending BDM to richer, multi-scale representations, remains an important direction for future work.

#### 6.4.3 Alternative interpretation: constraint-induced algorithmic compression

An alternative explanation to the discussion in Section 6.1, which we cannot verify within the scope of the present analysis, is that the observed reduction in BDM's relative advantage with increasing model capacity may not reflect convergence toward entropy *per se*, but rather an increased tendency toward memorization. Although theoretical considerations suggest such a trend, it remains unclear whether the model sizes explored here lie within a regime in which this effect dominates. Under this interpretation, greater flexibility in the parameter space could weaken the correspondence between structural compression and training loss. In particular, constraints such as weight binarization and limited model capacity may implicitly promote the compression of algorithmic structure, an effect that could diminish as these constraints are relaxed. Disentangling these factors would require further experiments, potentially involving full-precision networks with binary base expansions and larger computed CTM blocks, or improved approximations of algorithmic complexity capable of operating at larger scales.

## 7 Conclusion

This work presents a principled investigation of neural network training through the lens of algorithmic information theory. By applying the Block Decomposition Method (BDM) to Binarized Neural Networks (BNNs), we demonstrated that algorithmic complexity offers a more sensitive and stable indicator of training dynamics than traditional entropy.

Empirical results across multiple architectures show that BDM often correlates more strongly with training loss than entropy, particularly in smaller models, where algorithmic regularities are more apparent under BDM. These findings offer direct empirical support for the view that training in small binarized neural networks operates as a process of algorithmic compression, transforming random initializations into structured, compressible configurations that reflect the underlying data-generating process. Control experiments (shuffled-label MNIST and datasets with fully random inputs and labels) further reinforce this interpretation: in the absence of meaningful structure, the advantage of BDM disappears, and its behavior converges toward that of entropy. These results confirm that BDM captures structural features intrinsic to learning, beyond distributional statistics. Importantly, these findings are restricted to the regime of small binarized neural networks, where BDM provides a meaningful approximation of algorithmic complexity and remains sensitive to structural regularities beyond entropy. At the same time, we do not argue against the use of entropy-based measures: our analysis relies on the comparison of both measures, and entropy remains a useful and well-established baseline for statistical compression, both in theory and in practice.

We also emphasize that these results do not constitute a formal proof of algorithmic compression or a direct evaluation of description length in the Minimum Description Length (MDL) sense. Rather, they provide empirical evidence that, in the setting of small binarized MLPs, the shift toward more compressible representations emerges naturally, without an explicit compression objective.

Taken together, our results highlight the potential of algorithmic complexity measures to enrich our understanding of neural network behavior. They open new directions for future work, including the development of complexity-aware training regimes, regularization strategies based on algorithmic information theory, the design of learning systems that exploit causal-compressibility as a guiding principle, and systematic comparisons between BDM and other complexity measures, such as compression-based approaches (e.g., Lempel-Ziv complexity), graph-theoretic measures, and symmetry-based structural metrics, to better understand their complementary roles in characterizing learning dynamics, as explored in prior work on algorithmic and graph-theoretic complexity (Zenil et al., 2015a; Zenil, 2020). This broader perspective is especially relevant in the context of emerging architectures characterized by localized computation, such as sparse neural networks (Frankle and Carbin, 2019), transformers (Vaswani et al., 2017), Mixture-of-Experts (MoE) models (Shazeer et al., 2017), and Kolmogorov-Arnold Networks (KANs) (Liu et al., 2025). In these systems, BDM may have an even greater advantage, as it is particularly well-suited for characterizing modular structures (Hernández-Orozco et al., 2018).

Our work contributes to ongoing efforts to address a longstanding challenge: integrating algorithmic complexity and algorithmic probability, long proposed as a theoretical solution to AI through universal induction, into practical machine learning. Despite their foundational role in the theoretical foundations of artificial intelligence, these concepts have remained largely disconnected from neural network training. By operationalizing algorithmic complexity via BDM in binarized architectures, we take a step toward bridging this gap, replacing statistical proxies like entropy with causally grounded, algorithmic measures. In doing so, we contribute to realizing algorithmic theories of learning in practice, bringing foundational principles of AI closer to their application in modern machine learning.

## Data Availability

The raw data supporting the conclusions of this article will be made available by the authors, without undue reservation.
