# Supplementary material for: Binarized neural networks converge toward algorithmic simplicity: empirical support for the learning-as-compression hypothesis
Source: Front Comput Neurosci. 2026 May 29;20:1791546. doi: 10.3389/fncom.2026.1791546 (PMC13260101; doi:10.3389/fncom.2026.1791546)
Supplement: Supplementary file 1 [file Data_Sheet_1.pdf]

frontiers

Supplementary Material

1 TRAINING HYPERPARAMETERS

Table S1 summarizes the training hyperparameters used for each dataset, including learning rate, batch size, and early-stopping patience. Unless otherwise noted, hyperparameters were kept fixed across architectures within each dataset.

Table S1. Training hyperparameters used for each dataset. Patience refers to the number of epochs without validation improvement before early stopping is triggered. "N/A" indicates that early stopping was not applied.

Dataset	Learning rate	Batch size	Patience
mnist	0.001	128	5
fashion_mnist	0.001	128	5
sincos	0.001	64	5
uci_har	0.01	32	10
uci_har_raw	0.01	32	10
mnist_shuffled	0.001	128	N/A
random	0.001	128	N/A

2 COMPLETE EXPERIMENTAL RESULTS

In Table S2, we report the complete set of experimental results referenced in the main text, covering all datasets, model architectures, and training configurations evaluated.

Table S2. Complete experimental results across datasets and architectures. Reported values are correlation intervals [95% CI] between training loss and complexity metrics. Bold values indicate cases of non-overlapping confidence intervals, with the higher correlation highlighted. Δr and $\Delta \rho$ report the difference between midpoint estimates (midpoint(BDM) minus midpoint(entropy)), computed from the same rounded bounds. Accuracy is reported as mean $\pm$ standard deviation. Epochs denotes the average number of epochs used (mean $\pm$ std), and N denotes the number of valid runs used for bootstrap estimation.

Dataset	Hidden layers	Params	Entropy r	BDM r	Δr	Entropy ρ	BDM ρ	$\Delta \rho$	Accuracy (%)	Epochs	N
mnist	16	1760	[0.60, 0.68]	**[0.91, 0.93]**	0.28	[0.58, 0.68]	**[0.77, 0.85]**	0.18	79.5 ± 1.2	13.3 ± 5.2	199
mnist	80	8800	[0.84, 0.86]	**[0.92, 0.93]**	0.08	[0.83, 0.88]	**[0.89, 0.93]**	0.06	88.5 ± 0.4	20.5 ± 5.8	200
mnist	192	21120	[0.86, 0.88]	**[0.92, 0.92]**	0.05	[0.93, 0.95]	**[0.96, 0.97]**	0.03	90.3 ± 0.3	24.8 ± 6.2	200
mnist	8, 4	872	[0.47, 0.60]	**[0.77, 0.86]**	0.28	[0.52, 0.68]	**[0.74, 0.85]**	0.19	52.1 ± 5.3	9.3 ± 3.6	163
mnist	16, 8	1808	[0.55, 0.65]	**[0.90, 0.93]**	0.31	[0.58, 0.70]	**[0.85, 0.91]**	0.24	67.9 ± 2.4	11.2 ± 4.2	190
mnist	32, 16	3872	[0.74, 0.79]	**[0.92, 0.94]**	0.16	[0.73, 0.81]	**[0.84, 0.90]**	0.10	79.5 ± 1.0	14.0 ± 5.0	200
mnist	64, 32	8768	[0.84, 0.86]	**[0.93, 0.94]**	0.09	[0.85, 0.89]	[0.89, 0.92]	0.04	85.9 ± 0.5	17.5 ± 5.5	200
mnist	128, 64	21632	[0.86, 0.87]	**[0.92, 0.93]**	0.06	[0.92, 0.94]	[0.93, 0.95]	0.01	89.0 ± 0.4	20.3 ± 5.5	200
mnist	12, 24, 16	2032	[0.51, 0.63]	**[0.84, 0.90]**	0.30	[0.50, 0.63]	**[0.70, 0.80]**	0.19	69.4 ± 1.9	10.9 ± 4.1	194
mnist	40, 64, 32	8928	[0.77, 0.80]	**[0.91, 0.93]**	0.14	[0.71, 0.79]	**[0.80, 0.86]**	0.08	83.7 ± 0.7	16.1 ± 5.2	200
mnist	64, 128, 48	21216	[0.78, 0.81]	**[0.87, 0.89]**	0.08	[0.79, 0.84]	[0.81, 0.87]	0.03	87.0 ± 0.5	18.0 ± 5.7	200
mnist	12, 24, 16, 8	2080	[0.50, 0.62]	**[0.80, 0.87]**	0.27	[0.50, 0.64]	**[0.71, 0.81]**	0.19	61.3 ± 3.0	10.7 ± 4.4	191
mnist	32, 64, 44, 16	8928	[0.66, 0.73]	**[0.87, 0.90]**	0.19	[0.67, 0.76]	**[0.80, 0.86]**	0.11	79.4 ± 1.0	14.0 ± 5.0	200
mnist	64, 128, 48, 16	21664	[0.75, 0.80]	**[0.89, 0.91]**	0.12	[0.76, 0.83]	[0.82, 0.88]	0.06	83.2 ± 0.7	15.4 ± 5.2	200
fashion_mnist	8, 4	872	[0.07, 0.25]	[0.13, 0.36]	0.08	[0.14, 0.35]	[0.16, 0.39]	0.03	50.4 ± 4.9	10.5 ± 4.2	144
fashion_mnist	16, 8	1808	[-0.04, 0.11]	**[0.21, 0.38]**	0.26	[-0.06, 0.10]	**[0.20, 0.38]**	0.27	65.9 ± 2.1	13.0 ± 5.1	197
fashion_mnist	32, 16	3872	[0.03, 0.17]	**[0.30, 0.46]**	0.28	[0.04, 0.18]	**[0.32, 0.48]**	0.29	73.1 ± 0.9	15.0 ± 5.4	200
fashion_mnist	64, 32	8768	[0.09, 0.22]	**[0.26, 0.40]**	0.18	[0.09, 0.23]	**[0.30, 0.45]**	0.21	76.9 ± 0.6	17.4 ± 6.2	200
fashion_mnist	128, 64	21632	[0.20, 0.31]	[0.24, 0.37]	0.05	[0.24, 0.36]	[0.30, 0.43]	0.07	79.1 ± 0.5	18.3 ± 5.4	200
sincos	16	832	[0.18, 0.30]	**[0.60, 0.71]**	0.42	[0.25, 0.42]	**[0.61, 0.73]**	0.33	93.9 ± 0.9	17.5 ± 5.1	175
sincos	80	4160	[0.27, 0.40]	**[0.68, 0.75]**	0.38	[0.33, 0.47]	**[0.78, 0.85]**	0.41	97.0 ± 0.6	19.0 ± 7.2	200
sincos	192	9984	[0.34, 0.45]	**[0.66, 0.73]**	0.30	[0.45, 0.58]	**[0.80, 0.88]**	0.33	98.0 ± 0.5	20.2 ± 7.3	200
sincos	8, 4	456	[0.24, 0.39]	**[0.62, 0.75]**	0.37	[0.36, 0.58]	**[0.64, 0.78]**	0.24	83.3 ± 4.5	13.8 ± 4.7	125
sincos	16, 8	976	[0.23, 0.36]	**[0.67, 0.75]**	0.41	[0.31, 0.47]	**[0.69, 0.79]**	0.35	90.9 ± 1.8	15.7 ± 5.2	188
sincos	32, 16	2208	[0.25, 0.36]	**[0.63, 0.72]**	0.37	[0.30, 0.43]	**[0.64, 0.75]**	0.33	94.6 ± 1.1	19.5 ± 6.2	200
sincos	64, 32	5440	[0.36, 0.45]	**[0.71, 0.77]**	0.33	[0.45, 0.55]	**[0.77, 0.83]**	0.30	96.5 ± 0.7	22.0 ± 6.9	200
sincos	128, 64	14976	[0.46, 0.53]	**[0.73, 0.77]**	0.26	[0.58, 0.67]	**[0.82, 0.87]**	0.22	97.7 ± 0.5	23.9 ± 6.8	200
sincos	12, 24, 16	1312	[0.14, 0.28]	**[0.44, 0.58]**	0.30	[0.20, 0.37]	**[0.45, 0.60]**	0.24	92.4 ± 1.4	13.1 ± 4.9	187
sincos	40, 64, 32	6656	[0.05, 0.19]	**[0.39, 0.53]**	0.34	[0.07, 0.22]	**[0.42, 0.57]**	0.35	95.6 ± 0.8	15.2 ± 5.3	198
sincos	64, 128, 48	17600	[0.01, 0.16]	**[0.31, 0.44]**	0.29	[0.05, 0.20]	**[0.33, 0.47]**	0.28	96.8 ± 0.7	15.5 ± 5.4	200
sincos	12, 24, 16, 8	1408	[0.03, 0.20]	**[0.43, 0.59]**	0.40	[0.04, 0.23]	**[0.44, 0.60]**	0.39	89.9 ± 2.1	11.3 ± 4.7	180
sincos	32, 64, 44, 16	7168	[0.09, 0.24]	**[0.36, 0.50]**	0.27	[0.15, 0.30]	**[0.41, 0.55]**	0.26	94.2 ± 1.1	13.8 ± 5.4	199
sincos	64, 128, 48, 16	18240	[0.06, 0.21]	**[0.35, 0.49]**	0.28	[0.04, 0.19]	**[0.37, 0.51]**	0.33	95.6 ± 0.8	13.0 ± 5.3	198
uci_har	8, 4	4544	[0.68, 0.77]	[0.63, 0.74]	-0.04	[0.57, 0.68]	[0.51, 0.63]	-0.05	73.7 ± 6.5	16.1 ± 8.2	185
uci_har	16, 8	9152	[0.79, 0.84]	[0.73, 0.81]	-0.04	[0.59, 0.68]	[0.47, 0.59]	-0.10	88.4 ± 1.8	21.6 ± 10.2	200
uci_har	32, 16	18560	[0.87, 0.89]	[0.84, 0.88]	-0.02	**[0.69, 0.76]**	[0.59, 0.68]	-0.09	92.8 ± 0.9	25.0 ± 10.2	199
uci_har	64, 32	38144	[0.89, 0.90]	[0.90, 0.91]	0.01	[0.83, 0.87]	[0.78, 0.83]	-0.05	94.2 ± 0.7	25.3 ± 10.6	199
uci_har	128, 64	80384	[0.88, 0.89]	[0.89, 0.90]	0.01	[0.89, 0.92]	[0.86, 0.90]	-0.03	95.0 ± 0.6	25.8 ± 10.2	199
uci_har_raw	4, 16	4768	[0.57, 0.70]	[0.49, 0.64]	-0.07	[0.54, 0.66]	[0.46, 0.60]	-0.07	50.9 ± 2.6	17.5 ± 9.3	187
uci_har_raw	8, 16	9440	[0.81, 0.86]	[0.77, 0.83]	-0.03	[0.70, 0.78]	[0.65, 0.75]	-0.04	54.7 ± 2.4	20.4 ± 9.6	193
mnist_shuffled	16	1760	[0.80, 0.82]	[0.82, 0.84]	0.02	[0.61, 0.68]	[0.67, 0.74]	0.06	11.6 ± 4.7	14.0 ± 0.0	200
mnist_shuffled	32, 16	3872	[0.60, 0.66]	[0.65, 0.71]	0.05	[0.67, 0.75]	[0.67, 0.75]	0.00	11.2 ± 4.3	14.0 ± 0.0	200
mnist_shuffled	12, 24, 16	2032	[0.58, 0.62]	**[0.64, 0.68]**	0.06	[0.86, 0.90]	[0.83, 0.88]	-0.03	11.4 ± 3.6	14.0 ± 0.0	200
mnist_shuffled	40, 64, 32	8928	[0.29, 0.39]	**[0.44, 0.53]**	0.15	[0.51, 0.63]	[0.62, 0.73]	0.10	11.7 ± 3.4	14.0 ± 0.0	200
mnist_shuffled	32, 64, 44, 16	8928	[0.34, 0.44]	[0.40, 0.50]	0.06	[0.52, 0.64]	[0.53, 0.65]	0.01	11.0 ± 3.8	14.0 ± 0.0	200
random	16	1760	[0.81, 0.84]	[0.78, 0.82]	-0.02	[0.70, 0.78]	[0.73, 0.81]	0.03	10.3 ± 0.2	14.0 ± 0.0	200
random	32, 16	3872	[0.75, 0.80]	[0.74, 0.79]	-0.01	[0.48, 0.57]	[0.50, 0.61]	0.03	10.3 ± 0.3	14.0 ± 0.0	200
random	12, 24, 16	2032	[0.70, 0.73]	**[0.74, 0.77]**	0.04	[0.82, 0.86]	[0.79, 0.85]	-0.02	10.3 ± 0.3	14.0 ± 0.0	200
random	40, 64, 32	8928	[0.39, 0.49]	[0.44, 0.53]	0.04	[0.54, 0.66]	[0.48, 0.61]	-0.06	10.0 ± 0.3	14.0 ± 0.0	200
random	32, 64, 44, 16	8928	[0.39, 0.49]	[0.41, 0.50]	0.01	[0.48, 0.59]	[0.42, 0.54]	-0.05	10.3 ± 0.3	14.0 ± 0.0	200
